# Neurophysiological indices for split phenomena: correlation with age and sex and potential implications in amyotrophic lateral sclerosis

**DOI:** 10.3389/fneur.2024.1371953

**Published:** 2024-03-07

**Authors:** Stefano Zoccolella, Giammarco Milella, Alessia Giugno, Vito Devitofrancesco, Rosaria Damato, Ludovica Tamburrino, Salvatore Misceo, Marco Filardi, Giancarlo Logroscino

**Affiliations:** ^1^Neurology Unit, San Paolo Hospital, Azienda Sanitaria Locale (ASL) Bari, Bari, Italy; ^2^Center for Neurodegenerative Diseases and the Aging Brain, University of Bari Aldo Moro at Pia Fondazione “Card. G. Panico”, Tricase, Italy; ^3^Department of Translational Biomedicine and Neurosciences (DiBraiN), University of Bari Aldo Moro, Bari, Italy

**Keywords:** neurophysiology, split-hand, split-leg, split elbow sign, amyotrophic lateral sclerosis

## Abstract

**Background:**

Split phenomena (SP) are characterized by patterns of differential muscle wasting and atrophy, which are highly prevalent in amyotrophic lateral sclerosis (ALS) patients. Several neurophysiological indicators, including the split-hand index (SHI), split-leg index (SLI), and split-elbow index (SEI), have been proposed to assess SP. Nevertheless, their cutoff values and the impact of age and sex on these measures remain unclear.

**Methods:**

We prospectively collected neurophysiological data from 300 healthy adult subjects. The following indices were measured from compound muscle action potentials (CMAPs): SHI [abductor pollicis brevis (APB_cmap_) x first dorsal interosseous (FDI)_cmap_/adductor digiti minimi (ADM_cmap_)], SEI (BICEPS_cmap_/TRICEPS_cmap_), SLI (extensor digit brevis (EDB)_cmap_/abductor Hallucis (AH)_cmap_), and the neurophysiological ratios APB_cmap_ /ADM_cmap_ and FDI_cmap_/ADM_cmap_. Multiple linear regression analysis was used to investigate the association between age, sex, CMAPs, and neurophysiological indicators.

**Results:**

The median SHI was 10.4, with a median APB_cmap_/ADM_cmap_ ratio of 0.9 and a median FDI_cmap_/ADM_cmap_ ratio of 1.2. The median SEI was 1.6 (IQR:1.1–2.4) and the median SLI was 0.7 (IQR:0.5–1.0). Negative associations were observed between age, most of the CMAPs, and all the neurophysiological indices, except for SLI. The male subjects exhibited significantly higher CMAP values for the first dorsal interosseous (FDI), biceps, and SHI compared to the female participants.

**Conclusion:**

Our findings highlight the importance of age- and sex-adjusted normative data for SP indices, which could enhance their diagnostic accuracy and clinical utility in patients with ALS. The SL index appears to be the most reliable indicator, as it showed no significant association with age or sex.

## Background

A striking manifestation of amyotrophic lateral sclerosis (ALS) is the preferential dysfunction of certain groups of muscles over others, despite receiving the same innervation, particularly in the intrinsic hand, the elbow, and the foot muscles. This phenomenon is termed the “split phenomenon” (SP) (1).

The most well-characterized and the first reported SP is the split-hand (SH) sign, which is characterized by the predominant involvement of muscles of the lateral aspect of the hand compared to the hypothenar eminence muscles (2, 3). In ALS, a phenomenon known as the “split-leg” (SL) sign is characterized by more pronounced wasting of the extensor digitorum brevis (EDB) in the lower limbs compared to the abductor hallucis (AH) (3, 4). Finally, the biceps brachii muscles manifest more prominent weakness than the triceps brachii in ALS (5, 6), and this is termed the “split-elbow” (SE) sign.

The mechanisms underlying these dissociated patterns of muscle dysfunction in ALS remain unclear: while some authors have proposed that corticomotoneuronal projections contribute significantly to motor neurons innervating affected muscles (7), others have suggested lower motor neuron (LMN) dysfunction (8–12), primarily driven by axonal hyperexcitability (13, 14).

Over the years, several neurophysiological methods have attempted to assess and quantify the SP in patients with ALS. However, consensus remains elusive regarding which methods represent the most useful neurophysiological indicator of ALS (1).

The most widely adopted neurophysiological index for the SH sign is the split-hand index (SHI), which is calculated by multiplying the compound muscle action potential (CMAP) amplitude of the abductor pollicis brevis (APB) and first dorsal interosseous (FDI) muscles, and then dividing this product by the CMAP amplitude recorded over the adductor digiti minimi (ADM) (15). The SHI has exhibited good sensitivity and specificity in distinguishing ALS from ALS mimic disorders, albeit with variable cutoff values ranging from 5.2 to 10 (8, 16). In a recent meta-analysis, a cutoff value of 7.4 was proposed for the SHI in patients with a disease duration of 8–20 months (17). Several neurophysiological indices based on CMAP amplitudes of different muscles have been proposed for assessing the SH sign, with the most commonly used being APB_CMAP_/ADM_CMAP_ and FDI_CMAP_/ADM_CMAP_ (with cutoff values of 0.6 and 0.9, respectively) (1, 8, 14, 16, 18). Nonetheless, all the above-mentioned indices for the SH sign present several limitations, with the most important limitation being the confounding effect of aging on intrinsic hand amyotrophy (1). Indeed, in a recent study on healthy subjects, Pechirra et al. found that CMAPs of APB, FDI, and ADM decreased with age at approximately 0.8, 0.7, and 0.3 mV/year, respectively. Consistently, the SHI decreased by approximately 0.15 per year, indicating a strong age-dependence and supporting the notion that SH may be a physiological phenomenon (19).

The neurophysiological assessment of the split-leg phenomenon is commonly represented by the split-leg index (SLI), calculated as the ratio of peroneal and tibial CMAPs, recorded by EDB and AH, respectively, according to the formula: SLI_CMAP_ = EDB_CMAP_/AH_CMAP_ (3). The SLI demonstrated high sensitivity and specificity (with a cutoff value of 0.5) in differentiating ALS from mimic disorders, such as lumbar spondylosis (3).

Finally, a single neurophysiological study proposed a split-elbow index (SEI), which is calculated by dividing the CMAP amplitude of the biceps by the CMAP of the triceps muscle [SEI_CMAP_ = Biceps_CMAP_/Triceps_CMAP_ (20)].

To date, no study has explored the confounding effect of aging on either SLI or SEI. Additionally, while the impact of sex on nerve conduction studies has been extensively investigated (21–23), no studies have examined its role on all the indices mentioned in this study.

Based on these considerations, we conducted a neurophysiological study to investigate the contribution of both age and sex to the most widely adopted neurophysiological indicators for the split phenomena, namely, SHI, SEI, and SLI.

## Materials and methods

Between September 2022 and May 2023, we prospectively collected data from adult subjects who were referred to our electromyography (EMG) center for neurophysiological investigation of upper and/or lower limbs. The subjects underwent testing for possible articular/neuromuscular diagnoses, with clinical and neurophysiological results within the normal range based on our normative data (24). The exclusion criteria of the study are as follows: a history of radicular neck/back pain, diabetes mellitus, or carpal tunnel syndrome, conditions potentially affecting the peripheral nervous system, such as dysendocrinopathies, deficiencies in vitamin E, B12, and folic acid, hepatic and renal insufficiency, HIV, and connective tissue brain disorders. Additionally, subjects with abnormal median or ulnar sensory nerve action potentials, abnormal motor conduction velocity of the ulnar nerve across the elbow, and abnormal motor conduction velocity of the peroneal nerve across the fibular head were excluded. The study was approved by the ethics committee for Medical Research at Azienda Sanitaria Locale Lecce on 25 May 2017 (approval number 6) and was performed following the Helsinki Declaration and its later amendments. All subjects provided written informed consent.

### Neurophysiology evaluation

The participants underwent stimulation with supramaximal stimuli (1.5-fold maximal intensity) on their right median and ulnar nerves at the wrist and the elbow. Motor responses from the APB, FDI, and ADM muscles were recorded using bipolar surface electrodes (20 × 15 mm). The position of the stimulator on the ulnar nerve was kept constant for FDI and ADM studies. The surface recording electrode was confirmed by contraction and was placed over the muscle belly. The reference electrodes were placed over the proximal interphalangeal joint of the fifth finger for the ADM or the thumb interphalangeal joint for APB and FDI (25).

The right musculocutaneous and radial nerves were supramaximally stimulated at Erb’s point. For the biceps brachii, the recording electrode was positioned over the midpoint of the muscle belly on the anterior surface of the arm, while the reference electrode was placed over the biceps brachii tendon in the cubital fossa. For the triceps CMAP, the active electrode was placed over the long head of the muscle, and the reference electrode was placed over the olecranon point (20).

The right peroneal nerve was stimulated at the ankle, and the right tibial nerve was stimulated at the medial malleolus with supramaximal stimuli. The surface recording electrodes were placed over the middle of the belly of the EDB and AH muscles, while the reference electrode was placed on the tendon distally (26).

Nerve conduction studies (NCS) were conducted at skin temperatures above 32°C using an electro-diagnostic device and software (EDX EMG/evoked potential equipment, Nicolet, America). All neurophysiological studies were performed by the same neurophysiologist (S.Z.), who has extensive expertise in these techniques. To ensure maximal CMAP amplitude, the active electrode site (confirmed by contraction) was adjusted at least three times on each muscle in all NCS. Muscle relaxation was controlled by the device’s loudspeaker. We measured the baseline-to-peak CMAP amplitude of the largest motor response with a filter setting (20–10 kHz) and sensitivity for recording CMAP set to 5 mV/division. Concentric needle EMG examination was performed on first the dorsal interosseous, abductor pollicis brevis, biceps, triceps brachialis, quadriceps femoris, tibialis anterior, and gastrocnemius muscles to identify neurogenic damage (muscle fiber activity at rest, giant /polyphasic motor unit potentials, and reduced recruitment).

### Neurophysiological indices

For the purpose of this study, we calculated the SHI, defined by Shibuya et al. (27), as APB_CMAP_ /ADM_CMAP_ (28) and FDI_CMAP_ /ADM_CMAP_ (29).

The SEI _CMAP_ was defined as: Biceps_CMAP_/Triceps_CMAP_ (20) and SLI_CMAP_ as EDB_CMAP_/AH_CMAP_ (4).

### Statistical analysis

The data were analyzed using descriptive statistics (median, interquartile range [IQR]). The Shapiro–Wilk test was used to investigate the normality of data distribution.

The effect of age, sex, and their interaction on neurophysiological measures was investigated through a set of multiple linear regression analyses. The estimated coefficients were used to calculate the corrected scores for each variable.

Statistical analysis was performed using Jamovi (version 2.3.21.0). A *p*-value of <0.05 was considered statistically significant.

## Results

All the subjects (*n* = 300) underwent at least the neurophysiological evaluation of the intrinsic muscles of the hand. However, some of them refused to undergo the complete neurophysiological protocol. We performed neurophysiological evaluation in the arm muscles of 267 subjects and in the leg muscles of 241 subjects, with a median age of 61 years and a male/female ratio of 1:2.

The demographic and clinical features are presented in Table 1. Our cohort encompassed a wide range of ages, with a median of 61 years (IQR: 54–70) and a slight male predominance (60%). The distribution of sex across different age groups was reported in Supplementary Figure S1.

**Table 1:** Clinical and demographic characteristics of the study cohort.

	Median (IQR) or frequencies (n. of patients)	5% lower limit
Age (years)	61.0 (54.1–70.0) (*N* = 300)	-
Sex (M/F)	166/134 (*N* = 300)	-
APB_CMAP_ amplitude (mV)	8.7 (7.5–10.6) (*N* = 300)	6.0
ADM_CMAP_ amplitude (mV)	9.4 (8.1–11.0) (*N* = 300)	6.5
FDI_CMAP_ amplitude (mV)	10.0 (8.0–12.6) (*N* = 300)	6.1
Split Hand index	10.4 (8.2–12.9) (*N* = 300)	5.2
Neurophysiological index: APB_CMAP_ /ADM_CMAP_	0.9 (0.8–1.1) (*N* = 300)	0.6
Neurophysiological index: ADM_CMAP_ /FDI_CMAP_	1.2 (1.0–1.4) (*N* = 300)	0.6
Biceps_CMAP_ amplitude (mV)	8.1 (6.2–10.4) (*N* = 267)	3.3
Triceps_CMAP_ amplitude (mV)	5.7 (4.3–8.3) (*N* = 267)	2.7
Split Elbow index	1.6 (1.1–2.4) (*N* = 267)	0.7
EDB_CMAP_ amplitude (mV)	6.3 (5.3–8.0) (*N* = 241)	4.1
AH_CMAP_ amplitude (mV)	8.7 (6.7–11.2) (*N* = 241)	5.0
Split Leg Index	0.7 (0.5–1.0) (*N* = 241)	0.3

IQR, interquartile range; N, number; M, Male; F, Female; APB, Abductor Pollicis Brevis; ADM, Adductor Digiti Minimi; FDI, First Dorsal Interosseous; EDB, Extensor Digitorum Brevis; AH, Abductor Hallucis; CMAP, Compound Muscle Action Potentials.

**Figure 1:**
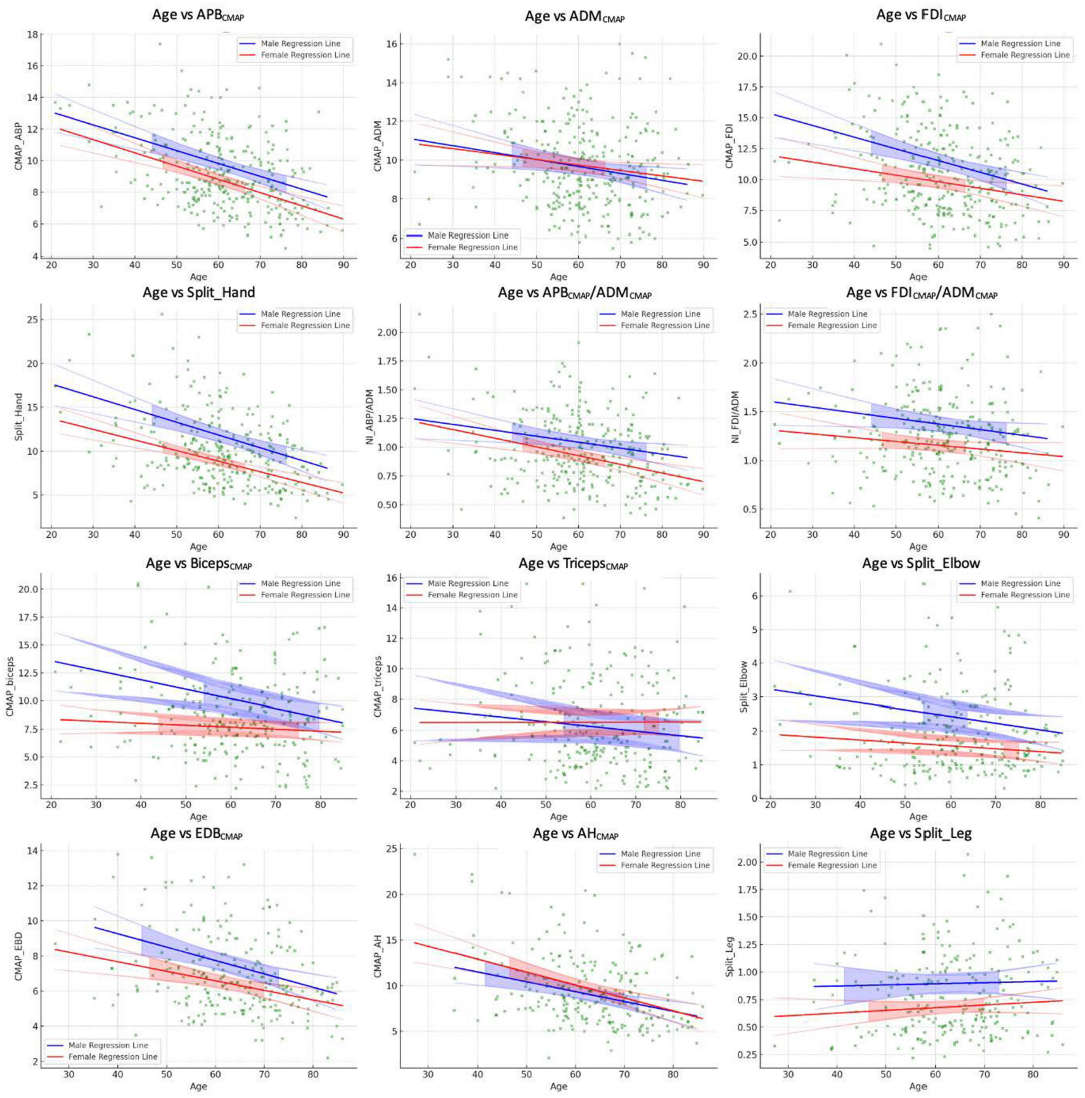
Linear regression findings of the age distribution of the neurophysiological measures.

Since the Shapiro–Wilk tests indicated significant deviations from normality for all variables (*p* < 0.001), we reported median values of the CMAP amplitude and neurophysiological indices in Table 1. The results of multiple regression analyses are shown in Table 2 and Figure 1.

**Table 2:** Multiple linear regression models of interaction between neurophysiological measures and age and sex.

Neurophysiological variables	Age	Age	Age	Sex	Sex	Sex	Age × sex	Age × sex	Age × sex
	β	95% CI	f2	β	95% CI	f2	β	95% CI	f2
APB_CMAP_^†^	−0.08^****^	[−0.11, −0.05]	0.1027	−0.86	[−3.22, 1.50]	0.0045	−0.002	[−0.04, 0.03]	0.0984
ADM_CMAP_^†^	−0.04^*^	[−0.06, −0.007]	0.1027	−0.38	[−2.87, 2.11]	0.0045	0.008	[−0.03, 0.05]	0.0984
FDI_CMAP_^†^	−0.09^****^	[−0.14, −0.05]	0.1027	−4.23^*^	[−7.83, −0.62]	0.0045	0.04	[−0.02, 0.01]	0.0984
Split_Hand^†^	−0.15^****^	[−0.19, −0.10]	0.1027	−4.44^*^	[−8.39–0.50]	0.0045	0.02	[−0.04, 0.09]	0.0984
APB_CMAP_/ADM_CMAP_^†^	−0.005^*^	[−0.009, −0.001]	0.1027	0.03	[−0.30, 0.36]	0.0045	−0.002	[−0.008, 0.003]	0.0984
FDI_CMAP_/ADM_CMAP_^†^	−0.006^*^	[−0.01, −0.0007]	0.1027	−0.33	[−0.77, 0.11]	0.0045	0.002	[−0.005, 0.009]	0.0984
Biceps_CMAP_^††^	−0.08^**^	[−0.13, −0.04]	0.0821	−6.52^**^	[−10.47, −2.58]	0.0669	0.07^*^	[0.004, 0.13]	0.0821
Triceps_CMAP_^††^	−0.03	[−0.07, 0.01]	0.0821	−1.59	[−5.27, 2.09]	0.0669	0.03	[−0.03, 0.09]	0.0821
Split_Elbow^††^	−0.02^*^	[−0.04, −0.004]	0.0821	−1.54^*^	[−2.90, −0.18]	0.0669	0.01	[−0.01, 0.03]	0.0821
EDB_CMAP_^†††^	−0.08^****^	[−0.11–0.04]	0.1027	−2.47	[−5.59, −0.64]	0.0045	0.02	[−0.03, 0.07]	0.0984
AH_CMAP_^†††^	−0.11^**^	[−0.17, −0.05]	0.1027	2.74	[−2.38, 7.86]	0.0045	−0.03	[−0.11, 0.05]	0.0984
Split_Leg^†††^	0.001	[−0.005, 0.007]	0.1027	−0.30	[−0.82, 0.21]	0.0045	0.001	[−0.01, 0.01]	0.0984

^*^p < 0.05.^†^Data available for 300 patients.^**^p < 0.01.^††^Data available for 267 patients.^***^p < 0.005.^†††^Data available for 241 patients.^****^p < 0.0005.APB, Abductor Pollicis Brevis; ADM, Adductor Digiti Minimi; FDI, First Dorsal Interosseous; EDB, Extensor Digitorum Brevis; AH, Abductor Hallucis; CMAP, Compound Muscle Action Potentials.

### Evaluation of the intrinsic hand muscles

The APB_CMAP_ showed a significant negative correlation with age (β = −0.08, *p* < 0.001). However, the associations with sex (β = −0.86, *p* = 0.47) and the interaction between age and sex (β = −0.002, *p* = 0.90) were not statistically significant (Table 2; Figure 1).

Similarly, ADM_CMAP_ showed a significant association with age (β = −0.04, *p* = 0.01) but not with sex (β = −0.38, *p* = 0.76) or age–sex interaction (β = 0.008, *p* = 0.71; Table 2; Figure 1).

The FDI_CMAP_ exhibited a significant negative relationship between both age (β = −0.09, *p* < 0.001) and sex (β = −4.23, *p* = 0.02). However, the interaction between age and sex was not statistically significant (β = 0.04, *p* = 0.15; Table 2; Figure 1).

Regarding the SHI, significant negative correlations were found between both age and sex (β = −0.017 and −05.93, respectively, *p* < 0.01). However, the interaction between age and sex was not significant (β = 0.04, *p* = 0.22; Table 2; Figure 1).

Neurophysiological ratios also showed significant negative associations between age and sex (β = −0.005 and −0.006, *p* = 0.008 and *p* = 0.03, respectively, Table 2; Figure 1). However, their associations between sex and the age–sex interaction were not significant.

### Evaluation of the arm muscles

The Biceps_CMAP_ showed significant associations between age, sex, and their interaction (β = −0.09, −6.52, and 0.07, all *p* < 0.05; Table 2; Figure 1). In contrast, the Triceps_CMAP_ was not significantly associated with age, sex, or their interaction (all *p* > 0.05).

SEI _CMAP_ showed a significant negative relationship between age and sex (β = −0.02 and −1.54, *p* = 0.02 and p = 0.03, respectively), but the age–sex interaction was not significant (β = 0.01, *p* = 0.31; Table 2; Figure 1).

### Evaluation of the leg muscles

Both EDB and AH CMAPs showed a significant negative correlation with age (β = −0.08 and β = −0.11, *p* < 0.001 and *p* = 0.001, respectively; Table 2; Figure 1). However, their associations with sex (EDB_CMAP_ β = −2.47, *p* = 0.12; AH_CMAP_ β = 2.74, *p* = 0.29), and the interaction between age and sex (EDB_CMAP_ β = 0.022, *p* = 0.37; AH_CMAP_ β = −0.03, *p* = 0.41) were not significant (Table 2; Figure 1).

The split-leg index did not exhibit significant associations with age, sex, or their interaction (all *p* > 0.05; Table 2; Figure 1).

## Discussion

In the present study, we examined the influence of age and sex on neurophysiological indicators of dissociated pattern of muscle wasting in a large cohort of adult participants with unremarkable neurological conditions. These neurophysiological indices play a critical role in the clinical and diagnostic work-up of patients with ALS and have been proposed as reliable measures for the differential diagnosis (1).

Our results build upon prior literature (4, 5, 26), which did not fully incorporate the potential impacts of age and sex. The dissociated wasting of the intrinsic hand, the elbow and the foot muscles represent typical manifestations of ALS, particularly in the initial stages when clinical signs are focal, especially for split-hand and split-leg signs (1, 8, 11). Conversely, the split-elbow remains actually poorly characterized (6).

Several studies have highlighted the role of SH and the SLI driven by CMAP (1, 11, 18); however, their diagnostic usefulness with validated cutoff values still remains debated in terms of specificity and sensitivity, due the potential and underestimated contributions of several confounding factors, including age (1, 19).

Our study demonstrated a significant correlation between advancing age and reduction in the CMAP amplitude for all the muscles evaluated, except for the triceps brachii muscle. Indeed, the CMAP amplitude of the triceps brachii showed no significant association with age. This pattern aligns with the general understanding of the neuromuscular system, wherein a decrease in the nerve potential amplitude is expected with increasing age (6, 21, 22, 30–33). These findings may be attributed to the effects of aging on nerve degeneration, including a reduction in the number and the size of axons as well as changes in the nerve fiber membrane (34).

Regarding the SHI, SEI and two neurophysiological ratios proposed to assess the SH sign, we found that these indices show negative correlations with age. These findings confirm and expand results of a previous study (19). In addition, the findings suggest that these neurophysiological ratios can represent specific patterns of differential muscle involvement in the normal aging process of the hand and the elbow muscles that go beyond the generalized age-related muscular atrophy.

Notably, this aging pattern may partially overlap with that commonly observed in the upper limbs of patients with ALS, potentially leading to an overestimation of the extent of neurodegeneration in elderly patients with ALS when such indices are employed. Consistent with this observation and in agreement with a previous study (11), the SHI decreased by 0.15 per year in our cohort of healthy individuals, while neurophysiological ratios such as APB/ADM, ADM/FDI, and SES decreased by 0.005, 0.006, and 0.02 per year, respectively.

The SLI was the only index that was not influenced by age, which makes it the most reliable neurophysiological indicator across all age groups. The reliability of the split-leg index across the age groups may reflect a similar decrease in the CMAP amplitude of both the AH and EDB muscles with advancing age (35, 36); therefore, their ratio (the split-leg index) remains relatively constant with aging. A previous study on the effect of age on the CMAP amplitude among healthy subjects found that the ratio between the median distal CMAP of the peroneal and tibial nerves is 0.3 in the age group of 15–34 years and 0.28 within the age group of 65–85 years (36).

Another noteworthy finding of our study is the significant contribution of sex to neurophysiological data. Specifically, sex significantly correlated with the CMAP amplitude of the FDI and biceps muscles, as well as with the indices, such as SHI and SEI, with male subjects showing higher CMAP amplitudes. This result is consistent with a recent study which observed that the FDI CMAP amplitude was significantly lower in male subjects compared to female subjects (25). Gender differences in specific muscles could be related to typically larger muscle mass and nerve fiber counts in male subjects, particularly in the biceps muscles (37–39). However, this association did not extend to other investigated muscles or indices, suggesting that the impact of sex on these variables may be muscle-specific, thereby complicating their interpretation further.

The strength of this study is that it is the first to systematically assess the proposed neurophysiological indicators for ALS-related split phenomena within a consistent cohort of subjects with unremarkable neurological conditions. However, the study has some limitations. The main limitation is that the patient population cannot be properly defined as “healthy” subjects, as they have had sufficient symptoms to be investigated with NCS/EMG. Therefore, even if the results of NCS were considered normal, we cannot exclude that their clinical condition could contribute to reduce their CMAPs. Another limitation is that we did not check for the confounding effect of laterality, which is particularly relevant in the radial and tibial nerves (40). However, we tried to limit such an effect by assessing all subjects on the right side. Nevertheless, it is worth noting that the inclusion of left-handed subjects, assessed by a validated clinical scale such as the Edinburgh Handedness Inventory (41), could have introduced some variability in our analysis. Another potential limitation could be the slight difference in the number of subjects who underwent all neurophysiological analyses of the upper and lower limbs.

In conclusion, the split-leg index seems to represent the most reliable indicator for SP, while our findings underline the necessity of considering age and sex as potentially confounding factors when interpreting the results of the neurophysiological data from the hands and the arms. Prospective, multi-center diagnostic evaluation according to the STARD criteria should be conducted on larger cohorts to create age- and sex-adjusted norms for these indices, thereby enhancing their diagnostic accuracy and validating their application in clinical contexts, especially in pathologies such as ALS.

## Data availability statement

The raw data supporting the conclusions of this article will be made available by the authors, without undue reservation.

## Ethics statement

The studies involving humans were approved by Azienda Sanitaria Locale Lecce. The studies were conducted in accordance with the local legislation and institutional requirements. The participants provided their written informed consent to participate in this study. Written informed consent was obtained from the individual(s) for the publication of any potentially identifiable images or data included in this article.

## Author contributions

SZ: Conceptualization, Investigation, Writing – original draft, Writing – review & editing. GM: Data curation, Formal analysis, Investigation, Methodology, Software, Supervision, Writing – original draft, Writing – review & editing. AG: Data curation, Investigation, Writing – original draft. VD: Data curation, Investigation, Writing – original draft. RD: Investigation, Writing – original draft. LT: Investigation, Software, Writing – original draft. SM: Conceptualization, Investigation, Writing – original draft. MF: Conceptualization, Data curation, Formal analysis, Investigation, Methodology, Software, Supervision, Validation, Writing – original draft, Writing – review & editing. GL: Funding acquisition, Project administration, Resources, Validation, Writing – original draft, Writing – review & editing.

## Supplementary material

Supplementary Figure S1 Distribution of sex across different age groups was reported.
