# Supplementary figures and images for: Neurophysiological indices for split phenomena: correlation with age and sex and potential implications in amyotrophic lateral sclerosis
Source: Front Neurol. 2024 Mar 7;15:1371953. doi: 10.3389/fneur.2024.1371953 (PMC10956616; doi:10.3389/fneur.2024.1371953)

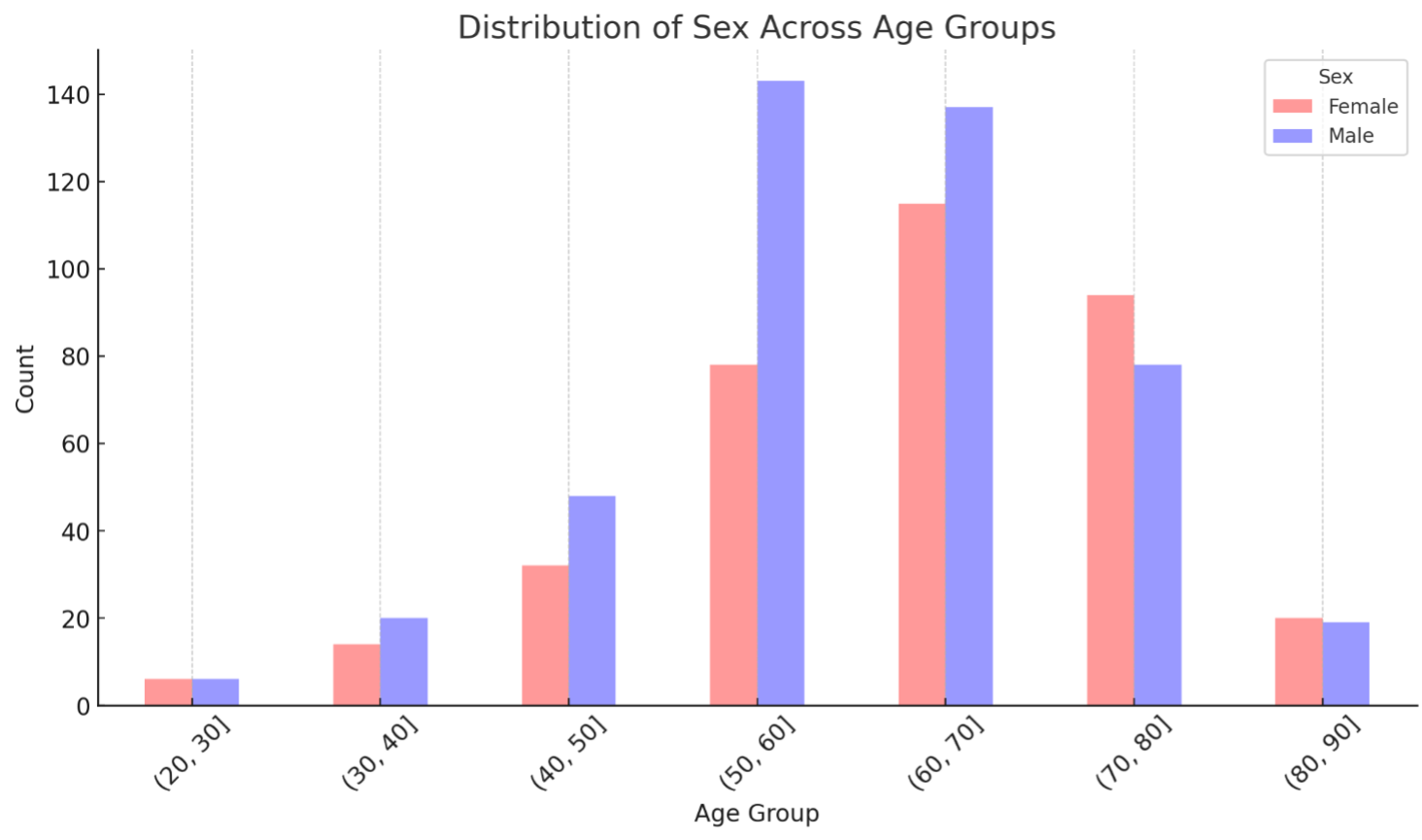

Supplement: Supplementary Figure S1 — Distribution of sex across different age groups was reported. [file Image_1.tiff]
